# Genetic Transformation in *Citrus*

**DOI:** 10.1155/2013/491207

**Published:** 2013-07-25

**Authors:** Dicle Donmez, Ozhan Simsek, Tolga Izgu, Yildiz Aka Kacar, Yesim Yalcin Mendi

**Affiliations:** ^1^Biotechnology Department, Institute of Applied and Natural Sciences, Çukurova University, 01330 Adana, Turkey; ^2^Horticulture Department, Agriculture Faculty, Çukurova University, 01330 Adana, Turkey; ^3^Horticulture Department, Agriculture Faculty, Ege University, 35100 İzmir, Turkey

## Abstract

Citrus is one of the world's important fruit crops. Recently, citrus molecular genetics and biotechnology work have been accelerated in the world. Genetic transformation, a biotechnological tool, allows the release of improved cultivars with desirable characteristics in a shorter period of time and therefore may be useful in citrus breeding programs. *Citrus* transformation has now been achieved in a number of laboratories by various methods. *Agrobacterium tumefaciens* is used mainly in citrus transformation studies. Particle bombardment, electroporation, *A. rhizogenes*, and a new method called RNA interference are used in citrus transformation studies in addition to *A. tumefaciens*. In this review, we illustrate how different gene transformation methods can be employed in different citrus species.

## 1. Introduction

*Citrus* species are the most widely grown fruit crops. Despite substantial genetic diversity and interspecific fertility, the genus *Citrus* includes some of the most difficult species to breed [1, 2]. This is due to several obstacles for conventional breeding. For example, most species are highly heterozygous and produce progeny that segregate widely for many characters when crosses are made. The juvenile periods are often very long, self- and cross-incompatibility and pollen and/or ovule sterility are relatively common, and the presence of adventitious somatic embryos in the nucellus of developing ovules of the most of *Citrus *greatly limits hybrid production [2, 3].

The genus *Citrus* possesses several undesirable characteristics including salt and cold sensitivity [4, 5]; they are also susceptible to diseases caused by fungi, bacteria and viruses, such as *Citrus* exocortis viroid (CEV), *Citrus* infectious variegation virus (CIVV), *Citrus* cachexia viroid (CCaV) and *Citrus* tristeza closterovirus (CTV) [5, 6]. Classical genetic selection, gene transfer, grafting, and micrografting techniques can contribute to the improvement of *Citrus* and propagation of selected species. Therefore, *in vitro* manipulation procedures leading to a rapid, direct bud regeneration for efficient micropropagation as well as genetic transformation are needed as a first step towards *Citrus* improvement. Practical benefits resulting from *in vitro* culture methods have already been reported in *Citrus* [5, 7, 8]. Recent developments in gene transfer techniques via the classical regeneration method have been applied to this genus and have opened the way to induce a specific genetic change within a period of time shorter than using the classical genetic selection method [5, 9, 10].

Conventional breeding methods have demonstrated limitations with respect to citrus improvement due to some of the biological characteristics of woody plants such as nucellar polyembryony, high heterozygosity, long juvenile period, and autoincompatibility [11, 12]. The development of biotechnological tools has made it possible to overcome some of these problems. In the specific case of citrus breeding programs, somatic hybridization [12–14] and genetic transformation [12, 15, 16] have been applied in many countries [10, 12, 17, 18].

In recent years, there has been a major thrust in citrus improvement as competition from international citrus markets, disease, and pest pressure and other abiotic and biotic stress conditions stimulate worldwide interest [19, 20]. Several strategies exist for the genetic improvement of citrus including conventional breeding and genetic transformation [20, 21]. Currently, genetic transformation of citrus as a tool for citrus improvement is gaining popularity. This method is especially useful in cases where it is not possible to introduce a particular trait of interest to another elite cultivar using conventional breeding. *Citrus* cultivars vary in their response to *in vitro* organogenesis and genetic transformation. This results in the need for cultivar-specific optimization of *in vitro* protocols [20, 22]. 

Among the several methods available for the genetic transformation of citrus, the most popular method to transform a wide range of citrus cultivars is *Agrobacterium*-mediated transformation using epicotyl explants as target cells for incorporation of the T-DNA [20, 23]. However, this method is not suitable for the transformation of any seedless cultivar. Also, special cultivars in the mandarin group remain robust to transform using this method [20, 22, 24]. 

## 2. Transformation Studies in *Citrus*

Genetic transformation and somatic hybridization studies are already integrated in *Citrus *breeding programs in several countries. Genetic transformation of *Citrus* is a promising tool that enables the introduction of desirable traits without altering the genetic background [25]. Genetic transformation of citrus has been reported, by using several methods (Table 1).

*Agrobacterium* has been the most frequently used genetic transformation method in *Citrus* with explants collected from seedlings germinated *in vitro* or under greenhouse conditions [68]. 

Transformation studies have been done for two decades in citrus. In the last few years, different transformation methods such as RNA silencing are used. In order to carry out successful gene transformation studies in citrus, optimized *in vitro* regeneration protocol is needed. Researchers should optimize efficient regeneration protocol before starting transformation studies. There are also many efficient regeneration protocols published in different citrus species.

Orbović et al. [36] investigated the effects of seed age on shoot regeneration potential and transformation rate of “Duncan” and “Flame” grapefruit cultivars, along with “Hamlin” sweet orange cultivar. Genetic transformation of citrus explants was carried out as previously described [93] using *A. tumefaciens* strain EHA105 [94] containing a binary vectors derived from pD35s [22]. In conclusion, the regeneration potential and transformability of citrus juvenile explants are different among cultivars and also change within the fruit harvest season. Because of these findings, especially the latter one, it will be extremely difficult to develop a universal protocol for genetic transformation of citrus. Optimal transformation efficiency will require flexible procedures that account for cultivar variability and timing of seed collection. In another study, a protocol was developed for regeneration of transgenic plants via *A. tumefaciens*-mediated transformation of leaf segments from “Valencia” sweet orange (*C. sinensis* L. Osbeck) using *gfp* (green fluorescence protein) as a vital marker [27]. The transformation methodology described by Khan et al. [27] was an important finding for generating transgenic plants using leaf segments as explants.

In addition to transformation studies via *A. tumefaciens*, recently, *A. rhizogenes* has been used. Many reports suggest the use of *A. rhizogenes* for expression of the rol genes and also to deliver foreign genes to susceptible plants [95]. The hairy root harbours the T-DNA segment of Ri-plasmid within its nuclear genomes [96]. *A. rhizogenes* are also capable of transferring the T-DNA of binary vectors *in trans*, thereby facilitating the selection of transgenic plants from screened hairy roots [95]. *A. rhizogenes*-mediated transformation system was found to be very useful in genetic manipulation of plants for the production of phytochemicals [97], large scale secondary metabolite production [98], monoclonal antibody production [99], and phytoremediation [100]. There are many reports that suggest the successful use of *A. rhizogenes* harbouring binary vectors with desired gene constructs [95] for plant genetic transformation [101]. Due to low transformation efficiency of *A. rhizogenes*, many researchers have worked to optimize transformation methods.

Chávez-Vela et al. [72] used *A. rhizogenes* A4 agropine-type strain to develop the transformation system. A4 contains wild-type plasmid pRi A4 which confers hairy-root genotype and binary vector pESC4. In the study seventy-five-day-old sour orange seedlings were used and transgenic sour orange (*C. aurantium* L.) plants were regenerated from *A. rhizogenes* transformed roots. 91% of explants produced transformed roots with an average of 3.6 roots per explant.

In another study transgenic Mexican lime (*C. aurantifolia* (Christm.) Swing) plants were regenerated from tissues transformed by *A. rhizogenes* strain A4, containing the wild-type plasmid pRiA4 and the binary vector pESC4 with nos-npt II and cab-gus genes. More than 300 Mexican lime transgenic plants were obtained, 60 of which were adapted to growing in soil [2].

In addition to the indirect gene transfer methods, there are studies performed by direct gene transfer methods in citrus. Bespalhok Filho et al. [69] carried out to optimize the conditions for transient gene expression through particle bombardment on Carrizo citrange (*C. sinensis*  ×  *Poncirus trifoliata*) thin epicotyl sections. The best conditions for transient GUS expression were M-25 tungsten particles, 1550 psi helium pressure, 9 cm distance between specimen, and DNA/particle holder and culture of explants in a high osmolarity medium (0.2 M  mannitol + 0.2 M sorbitol) 4 h prior and 20 h after bombardment. Under these conditions, an average of 102 blue spots per bombardment (20 explants/plate) were achieved. It is stated that protocol is currently being used for transformation of Carrizo citrange and sweet orange (*C. sinensis*). 

Electroporation is an effective direct gene transfer system used for citrus transformation. Hidaka and Omura [90] used electroporation methods for gene transformation in citrus. Protoplasts were prepared from embryogenic callus of “Ohta” ponkan (*C. reticulata* Blanco) and electroporation with exponential decay pulses was carried out in the solution containing the *β*-glucuronidase (GUS) chimeric gene coupled to the CaMV 35S promoter (pBI221). At 24 hr after incubation, significant GUS activity was detected in the cells by fluorometric assay. Another alternative method for direct gene transformation had been developed in sweet orange (*C. sinensis* (L.) Osbeck). Plasmid DNA encoding the nondestructive selectable marker enhanced green fluorescent protein gene was introduced using polyethylene glycol into protoplasts of “Itaborai” sweet orange isolated from an embryogenic nucellar-derived suspension culture. Following protoplast culture in liquid medium and transfer to solid medium, transformed calluses were identified via expression of the green fluorescent protein, physically separated from nontransformed tissue and cultured on somatic embryogenesis induction medium. Transgenic plantlets were recovered from germinating somatic embryos and by *in vitro* rooting of shoots [82].

As well as the transformation studies conducted for gene expression, several studies conducted for gene silencing. RNA interference (RNAi) are a posttranscriptional gene-silencing phenomenon induced by double-stranded RNA. It has been widely used as a knockdown technology to analyze gene function in various organisms. Although RNAi was first discovered in worms, related phenomena such as posttranscriptional gene silencing and coat protein-mediated protection from viral infection had been observed in plants prior to this. In plants, RNAi is often achieved through transgenes that produce hairpin RNA. For genetic improvement of crop plants, RNAi has advantages over antisense-mediated gene silencing and cosuppression, in terms of its efficiency and stability [102]. Soler et al. [32] stated Citrus tristeza virus (CTV), the causal agent of the most devastating viral disease of citrus, has evolved three silencing suppressor proteins acting at intra- (p23 and p20) and/or intercellular level (p20 and p25) to overcome host antiviral defence. Mexican lime was transformed with an intron-hairpin vector carrying full-length, untranslatable versions of the genes p25, p20, and p23 from CTV strain T36 to silence the expression of these critical genes in CTV-infected cells. Three transgenic lines presented complete resistance to viral infection, with all their propagations remaining symptomless and virus-free after graft inoculation with CTV-T36, either in the nontransgenic rootstock or in the transgenic scion. Accumulation of transgene-derived siRNAs was necessary but not sufficient for CTV resistance. Inoculation with a divergent CTV strain led to partially breaking the resistance, thus showing the role of sequence identity in the underlying mechanism. Results are a step forward to developing transgenic resistance to CTV and also show that targeting simultaneously by RNA interference (RNAi) the three viral silencing suppressors appear critical for this purpose, although the involvement of concurrent RNAi mechanisms cannot be excluded.

## 3. Conclusion

Genetic transformation is an attractive alternative technique for citrus genetic improvement. However, transformation efficiencies are generally low, and protocols are dependent on species, or even cultivar dependent. One of the limitations within this technology is low plant regeneration frequencies especially for many of the economically important citrus species [65]. In addition, difficulty in rooting transgenic shoots for some citrus cultivars has been reported [10, 89, 91]. Development of effective genetic transformants therefore requires specific studies on *in vitro* regeneration conditions for each genotype.

The development of direct genetic manipulation techniques has provided new opportunities for plant improvement. Plant transformation has made it possible to modify just one or two traits, while retaining the unique characteristics of the original cultivar. The characters that could potentially be manipulated by genetic transformation of *Citrus* include pest and disease resistance, growth habit, and fruit quality. In order to use this technology, it is essential to develop efficient genetic transformation systems for *Citrus*. [2].

## Figures and Tables

**Table 1:** Transformation researches in citrus.

Species	Transferred genes	Transformation method	References
*C. sinensis* L. Osb.	*GUS* and *npt*II	*A. tumefaciens *	[26]
*C. sinensis* L. Osb.	*gfp *	*A. tumefaciens *	[27]
*C. sinensis* L. Osb.	*GUS *	*A. tumefaciens *	[28]
*C. sinensis *L. Osb. and Carrizo citrange	*uid*A, *npt*II	*A. tumefaciens *	[29]
*C. paradisi *Macf.	*RdRp*, *Gfp*, and *Gus *	*A. tumefaciens *	[30]
*C. sinensis *L. Osb.	*CTV-CP *	*A. tumefaciens *	[31]
*C. aurantifolia *	*p25*, *p20*, and *p23 *	RNA interference	[32]
*C. aurantifolia *Swingle	*AtSUC*2*, RSs*1*, RTBV, GUS*, *rol*C	*A. tumefaciens *	[33]
*C. paradisi *	*att*E	*A. tumefaciens *	[34]
*C. unshiu* Marc	*miraculin *	*A. tumefaciens *	[35]
*C. sinensis *L. Osbeck and *C. paradisi* Macf.	*GFP *	*A. tumefaciens *	[36]
*C. sinensis *L.	*CTV-GFP *	*A. tumefaciens *	[37]
*C. sinensis *Osb.	*Shiva* A and *Cecropin* B	*A. tumefaciens *	[38]
*C. sinensis *	CPsV *cp* (ihpCP), *54K* (ihp54K), and *24K *(ihp24K)	*A. tumefaciens* RNA silencing	[39]
*C. sinensis *L. Osb.	*GFP *and *npt*II	*A. tumefaciens *	[40]
*C. sinensis *L. Osb.	*pth*A*-nls *	*A. tumefaciens *	[41]
*Poncirus trifoliata *L. Raf.	*AhBADH *	*A. tumefaciens *	[42]
*C. sinensis *L. Osb.	*Cy-GFP* and *Er-GFP *	*A. tumefaciens *	[43]
*C. aurantifolia *Swingle	*gus-egfp *	*A. tumefaciens *	[44]
Tetraploid citrus rootstock selection “Orange #16”	*egfp*-*npt*II	*A. tumefaciens *	[45]
Carrizo citrange	*man*A and *egfp *	*A. tumefaciens *	[22]
*C. sinensis*, *C. reticulata C. amblycarpa* and *C. depressa *	*npt*II,* hpt*II,* and GFP *	*A. tumefaciens *	[20]
Carrizo citrange and *C. sinensis *L. Osb.	*Gfp *	*A. tumefaciens *	[46]
Carrizo citrange, *C. paradisi* Macf., *C. aurantifolia* Swingle *C. sinensis* L. Osb.	*EGFP *	*A. tumefaciens *	[23]
*“*Swingle” citrumelo and *C. sinensis* L. Osb.	*GUS* and *npt*II	Sonication-assisted *A. tumefaciens *(SAAT)	[47]
*C. sinensis *cv. Hamlin	*hrp*N	*A. tumefaciens *	[48]
*Poncirus trifoliata *[L.] Raf.	*uid*A and *npt*II	*A. tumefaciens *	[49]
*Poncirus trifoliata* [L.] Raf	*GFP *and *MAC12.2 *	*A. tumefaciens *	[50]
Carrizo citrange and *C. sinensis* L. Osb.	*uid*A and iaaM/H marker genes	*A. tumefaciens *	[51]
*C. sinensis* L. Osb.	*cp* and *nos* genes	*A. tumefaciens *	[52]
*C. sinensis* L. Osb.	Nospro-nptII-Noster	*A. tumefaciens *	[53]
Carrizo citrange and *C. sinensis* L. Osb.	*ipt* gene	*A. tumefaciens *	[54]
*C. paradise* Macf.	*CTV*-derived candidate resistance	*A. tumefaciens* RNA-mediated resistance	[55]
*Poncirus trifoliata* L. Raf.	*gfp *	*A. tumefaciens *	[56]
Carrizo citrange	*npt*II	*A. tumefaciens *	[57]
*C. aurantium, C. macrophylla, C. limon *and Troyer citrange	*CTV*-*p61* and *p23U *	*A. tumefaciens *	[58]
*C. sinensis* L. Osb.	*att*A	*A. tumefaciens *	[59]
*C. limonia *Osb.	*bO *	*A. tumefaciens *	[60]
*C. paradisi *	*RdRp *	*A. tumefaciens *	[61]
*C. jambhiri *Lush	*GUS* and *npt*II	*A. tumefaciens *	[62]
*C. sinensis* L. Osb.	*gfp* and *pme *	PEG	[63]
Swingle citrumelo	*uid*A, *npt*II, and *GUS *	*A. tumefaciens *	[64]
Carrizo citrange	*GUS* and *npt*II	*A. tumefaciens *	[65]
Carrizo citrange and* C. aurantifolia *	*GUS, GFP*, and *npt*II	*A. tumefaciens *	[66]
Carrizo citrange	Citrus blight-associated	*A. tumefaciens *	[67]
*C. sinensis* and *C. limonia *	*GUS *	*A. tumefaciens *	[68]
Carrizo citrange	*uid*A and *npt*II	Particle bombardment	[69]
*C. sinensis *	*pTA29-barnase *	*A. tumefaciens *	[70]
*Citrus sinensis *	*PMI *	*A. tumefaciens *	[12]
*Citrus sinensis *	*GUS* and *npt*II	*A. tumefaciens *	[71]
*Citrus aurantium *L.	*GUS* and *npt*II	*A. rhizogenes *	[72]
*Citrus paradisi *Macf.	*cp* and GUS	*A. tumefaciens *	[73]
*C. sinensis* L. Osb.	*GUS *	Electroporation	[74]
Carrizo citrange and *C. sinensis* L. Osb.	*GUS *	*A. tumefaciens *	[75]
*Citrus sinensis *L. Osbeck	*GUS* and *npt*II	*A. tumefaciens *	[18]
*C. reticulata *Blanco	*pTA29-barnase *	*A. tumefaciens *	[76]
*C. paradisi* Macf.	Carotenoid biosynthetic genes	*A. tumefaciens *	[16]
Carrizo citrange	*LFY* and *AP1 *	*A. tumefaciens *	[77]
*C. aurantium *L.	*cp *	*A. tumefaciens *	[78]
*C. paradisi* Macf.	*CP* and *T36 *	*A. tumefaciens *	[79]
Troyer citrange	*Bar* and *uid*A	*A. tumefaciens *	[80]
*C. aurantifolia *Swing.	*cp *	*A. tumefaciens *	[81]
*C. sinensis *(L.) Osb.	*Gfp *	PEG	[82]
*C. aurantifolia * Swing.	*GUS *	*A. tumefaciens *	[83]
*C. paradisi *Macf.	*GUS, uncp, gna *	*A. tumefaciens *	[84]
Carrizo citrange	*uid*A and *npt*II	*A. tumefaciens *	[85]
*C. sinensis *L. Osb.	*GUS *	*A. tumefaciens *	[86]
*C. aurantifolia* (Christm.) Swing.	*GUS* and *npt*II	*A. rhizogenes *	[2]
*C. aurantium *L.	*cp *	*A. tumefaciens *	[10]
Tangelo	*GUS* and *npt*II	Particle bombardment	[87]
Carrizo citrange	*GUS* and *npt*II	*A. tumefaciens *	[88]
*C. sinensis* L. Osb.	*GUS* and *npt*II	*A. tumefaciens *	[89]
*C. reticulata *Blanco	*GUS *	Electroporation	[90]
*Citrus *spp.	*GUS* and *npt*II	*A. tumefaciens *	[91]
*Citrus *spp.	*cat* and *npt*II	PEG	[92]
